# Gene panel analysis of 119 index patients with suspected periodic paralysis in Japan

**DOI:** 10.3389/fneur.2023.1078195

**Published:** 2023-01-26

**Authors:** Jun-Hui Yuan, Yujiro Higuchi, Akihiro Hashiguchi, Masahiro Ando, Akiko Yoshimura, Tomonori Nakamura, Yu Hiramatsu, Yusuke Sakiyama, Hiroshi Takashima

**Affiliations:** Department of Neurology and Geriatrics, Graduate School of Medical and Dental Sciences, Kagoshima University, Kagoshima, Japan

**Keywords:** periodic paralysis, *CACNA1S*, *SCN4A*, *KCNJ2*, gene panel sequencing

## Abstract

**Introduction:**

Genetic factors are recognized as the major reason for patients with periodic paralysis. The goal of this study was to determine the genetic causes of periodic paralysis in Japan.

**Methods:**

We obtained a Japanese nationwide case series of 119 index patients (108 men and 11 women) clinically suspected of periodic paralysis, and a gene panel analysis, targeting *CACNA1S, SCN4A*, and *KCNJ2* genes, was conducted.

**Results:**

From 34 cases, 25 pathogenic/likely pathogenic/unknown significance variants were detected in *CACNA1S* (nine cases), *SCN4A* (19 cases), or *KCNJ2* (six cases), generating a molecular diagnostic rate of 28.6%. In total, seven variants have yet been found linked to periodic paralysis previously. The diagnostic yield of patients with hypokalemic and hyperkalemic periodic paralyzes was 26.2 (17/65) and 32.7% (17/52), respectively. A considerably higher yield was procured from patients with than without positive family history (18/25 vs. 16/94), onset age ≤20 years (24/57 vs. 9/59), or recurrent paralytic attacks (31/94 vs. 3/25).

**Discussion:**

The low molecular diagnostic rate and specific genetic proportion of the present study highlight the etiological complexity of patients with periodic paralysis in Japan.

## 1. Introduction

Periodic paralysis (PP) is a rare skeletal muscle channelopathy induced by abnormal excitability of the sarcolemma, leading to episodes of flaccid paralysis in the extremities of patients. Symptoms commonly appear in the first or second decade, usually upon awakening in the middle of the night or early morning, and last for hours (occasionally days) before gradually disappearing. The calculated minimum point prevalence rates of PP have been reported at 0.38–0.69/100,000 in the UK and the Netherlands (1, 2).

Clinically, patients with PP with decreased serum potassium level (<3.5 mmol/L) during the paralytic attacks are subtyped as hypokalemic periodic paralysis (hypoPP), genetically linked to the mutations in *CACNA1S* (encoding α1-subunit of the skeletal muscle L-type calcium channel Cav1.1; hypoPP1) or *SCN4A* (encoding α1-subunit of voltage-gated sodium channel Nav1.4; hypoPP2) (3, 4). However, naming of the subtype of patients with PP with normal-range or high serum potassium levels is controversial, and in the present study, we refer to multiple recent publications and group these patients with serum potassium level ≥3.5 mmol/L as hyperkalemic periodic paralysis (hyperPP) (1, 2, 5, 6). *SCN4A* is the causative gene of hyperPP as well (7), and it is also responsible for *SCN4A*-related non-dystrophic myotonia, characterized by a heterogeneous phenotypic spectrum of myotonia (8, 9). Furthermore, mutations in the *KCNJ2* gene (encoding inward-rectifier potassium channel Kir2.1), which have been linked to Andersen-Tawil syndrome (ATS), could also result in a PP phenotype, although typically accompanied by ventricular arrhythmias and dysmorphism (10).

To date, large-group genetic studies concerning both hypoPP and hyperPP are inadequate, and only a few studies have covered all three abovementioned genes, *CACNA1S, SCN4A*, and *KCNJ2*. The genetic diagnostic rate of overall patients with PP remains unclear, which is estimated to be 64.1% in the USA or 56.6% in China (11, 12). In this study, among 119 index patients with PP, referring to broad diagnostic criteria, we present a low molecular diagnostic rate in Japan and reassess multiple clinical features associated with the diagnostic yield.

## 2. Materials and methods

### 2.1. Sample collection

**Figure 1:**
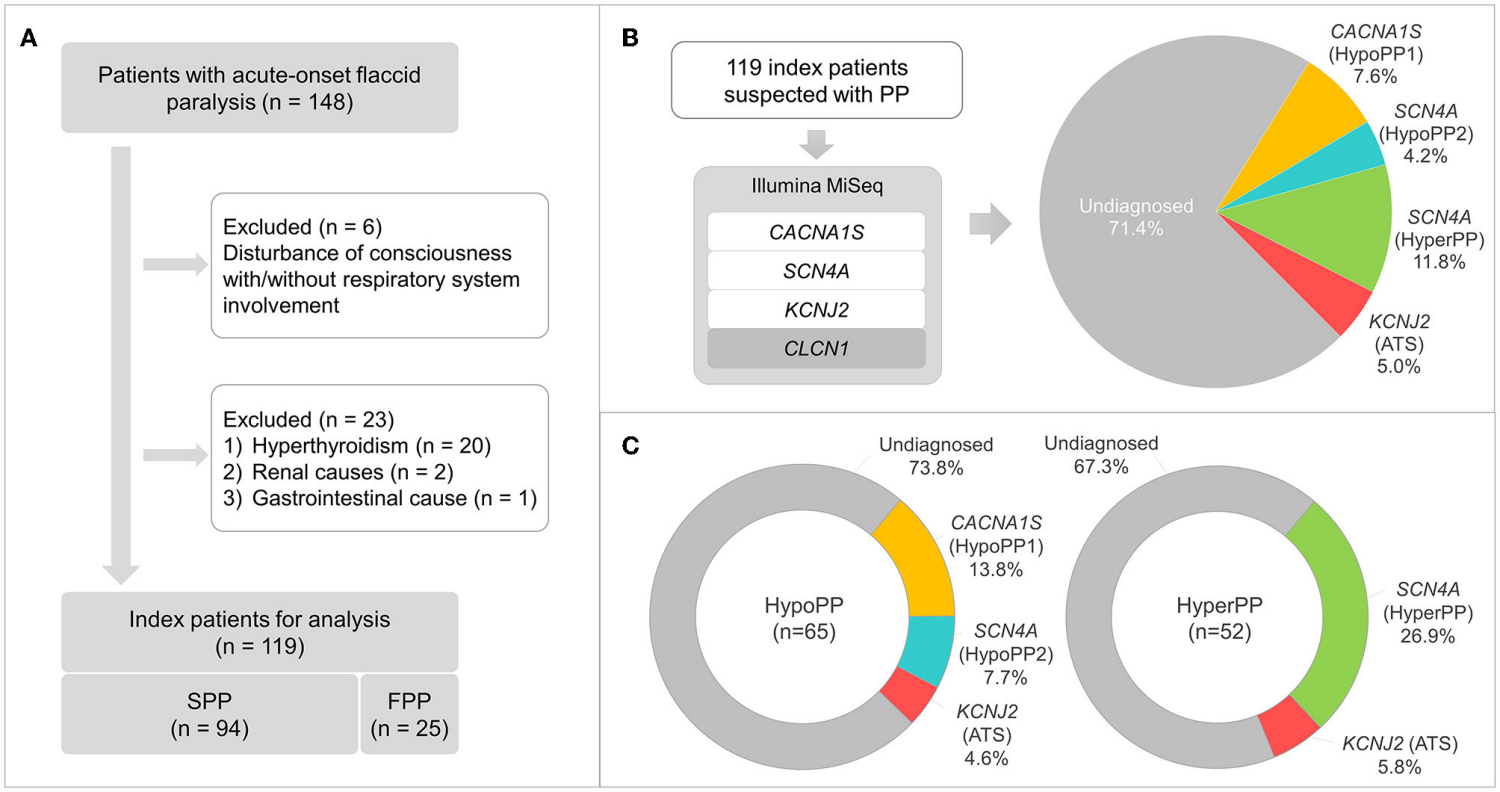
Patient selection flowchart, gene panel sequencing workflow, and findings. **(A)** Inclusion and exclusion criteria of patients clinically suspected with periodic paralysis (PP), and 119 index patients, consisting of 94 and 25 cases with sporadic (SPP) and familial PP (FPP), respectively, are selected for the following analysis. **(B)** Gene panel sequencing with Illumina MiSeq demonstrates a molecular diagnostic rate of 28.6%, and the genetic proportions are presented. **(C)** Genetic proportions of hypoPP (*n* = 65) and hyperPP (*n* = 52) patients.

This is a monocentric retrospective study that included a nationwide case series of 148 patients clinically suspected of PP in Japan (ranging from January 1999 to January 2022). All patients were examined by their attending doctors from the departments of neurology/pediatrics of local hospitals and then referred to our laboratory for genetic testing. The included criteria are acute-onset flaccid paralysis that resolves spontaneously or with potassium treatment within hours or days, without disturbance of consciousness and respiratory muscle involvement. Patients with hyperthyroidism, renal diseases (primary aldosteronism or IgA nephropathy), or gastrointestinal disorders were exempted. Ultimately, we collected 119 consecutive unrelated index patients in this project. Therein, 25 cases with more than one affected individual in their pedigrees were grouped as familial PP (FPP); 94 cases without any positive family history were grouped as sporadic PP (SPP). Within the FPP, 21 pedigrees were considered as autosomal dominant inheritance, encompassing more than one affected individual from ≥2 generations, while the inheritance pattern was not clear in the other four pedigrees. The inclusion and exclusion flowchart are illustrated in Figure 1A.

This research was authorized by the institutional review board of Kagoshima University (Application ID: 490). All patients/parents and their available family members provided informed consent for their participation in this study.

### 2.2. Genomic DNA isolation and Sanger sequencing

Blood samples were collected from patients and any of their available family members. Genomic DNA was extracted from peripheral blood lymphocytes using DNA extraction kits following the corresponding manufacturer's protocols. For DNA samples collected before 2013, Sanger sequencing was conducted on the voltage–sensor coding exons of *CACNA1S* and *SCN4A*, as well as the coding region of the *KCNJ2* gene, according to the initially published procedures (13).

### 2.3. Gene panel sequencing on Illumina MiSeq

All samples, whether previously sequenced by Sanger sequencing or obtained after 2013, were subjected to NGS-based gene panel sequencing. Primers were designed using the Primer 3 program, covering all coding exons and exon–intron junctions of *CACNA1S* (NM_000069.3), *SCN4A* (NM_000334.4), *KCNJ2* (NM_000891.3), and *CLCN1* (NM_000083.3). After multiplex polymerase chain reaction (PCR) (Qiagen Multiplex PCR Kit; Qiagen GmbH, Hilden, Germany), amplicons were pooled together and sequenced on the Illumina MiSeq platform (Figure 1A). Low coverage amplicons (reading depth <10) and suspected variants were subsequently substantiated by Sanger sequencing.

### 2.4. Variant annotation and interpretation

Sequencing data alignment to human reference genome GRCh37, variant processing/annotation, and analysis were conducted *via* CLC Genomics Workbench (Qiagen, Hilden, Germany), Ensembl-VEP, and in-house R scripts. All variants were contrasted against two population databases, including the East Asian population in Genome Aggregation Database (gnomAD_EAS v2.1.1; https://gnomad.broadinstitute.org) and the Japanese Multi Omics Reference Panel (jMorp; https://jmorp.megabank.tohoku.ac.jp/202102/), as well as the Human Gene Mutation Database (HGMD 2022.2, Qiagen). In total, five *in silico* prediction scores were enrolled using dbNSFP (v4.0), consisting of SIFT, PolyPhen2, PROVEAN, FATHMM, and Condel (14). All suspected variants were interpreted using a modified American College of Medical Genetics and Genomics/Association for Molecular Pathology (ACMG/AMP) classification and ClinGen Expert Panel consensus approaches (15, 16) (Supplementary Table 1). Only pathogenic (P), likely pathogenic (LP), and variant of unknown significance (VUS) variants are described here.

### 2.5. Statistical analysis

To contrast the frequencies for categorical variables, a two-tailed Fisher's exact test was conducted using a GraphPad online tool (https://www.graphpad.com/quickcalcs/contingency1/). A *p*-value of <0.05 was deemed substantial. The odds ratio and significance values of the variants in jMorp (ToMMo 38KJPN) were calculated using MedCalc (https://www.medcalc.org/calc/odds_ratio.php).

## 3. Results

### 3.1. Clinical analyses

Among 119 index cases with suspected PP, male and female patients accounted for 108 and 11, respectively. Based on the serum potassium levels during attacks, these patients were classified as hypoPP (65 cases) and hyperPP (52 cases), and two cases lacked serum potassium records. Approximately half of these patients had their first paralytic attack at the age ≤20 years (57 cases), while the other half had their onset age >20 years (59 cases). No onset record was available from three patients. There were 94 cases with two or more paralytic attacks and 25 cases with only one attack before the genetic screening. Clinical data of all patients are summarized in Supplementary Table 2.

### 3.2. Genetic findings

P/LP/VUS variants in the *CACNA1S* (nine cases), *SCN4A* (19 cases), or *KCNJ2* (six cases) genes were discovered in 34 cases, generating a detection rate of 28.6%. All these variants are listed in Table 1 along with their classification basis. Within 19 cases carrying *SCN4A* variants, respectively, five and 14 cases had hypoPP and hyperPP phenotypes. The diagnosed patients' detailed genetic proportions were 7.6 (hypoPP1), 4.2 (hypoPP2), 11.8 (hyperPP), and 5.0% (ATS) (Figure 1B). No P/LP/VUS variants were found in the *CLCN1* gene.

**Table 1:** All variants detected from 34 Japanese patients with PP.

**Gene**	**Nucleotide**	**Amino acid**	**gnomAD_EAS**	**jMorp**	**Modified ACMG/AMP guideline**	**Class**
*CACNA1S*	1582C > G	R528G	0	0	PS3 + PS4 + PM1 + PM2 + PM5(S) + PP3 + PP4	P
*CACNA1S*	1583G > A	R528H	0	0	PS3 + PS4 + PM1 + PM2 + PM5(S) + PP3 + PP4	P
*CACNA1S*	2700G > C	R900S	0	0	PS3 + PS4 + PM1 + PM2 + PM5 + PP3 + PP4	P
*CACNA1S*	3716G > A	R1239H	0	0	PS3 + PS4 + PM1 + PM2 + PM5 + PP3 + PP4	P
*CACNA1S*	3726G > T	R1242S	0	0	PS4(P) + PM1 + PM2 + PM5 + PP3 + PP4	LP
*SCN4A*	109G > A	A37T^†^	0.0003346	0.000245	PS4(M) + PP3 + PP4	VUS
*SCN4A*	664C > T	R222W	0	0	PS3 + PS4 + PM1 + PM2 + PM5 + PP3 + PP4	P
*SCN4A*	791T > C	F264S^†^	0	0	PS4(P) + PM2 + PP3 + PP4	VUS
*SCN4A*	1354G > A	E452K	0	0	PS4(P) + PM2 + PP3 + PP4	VUS
*SCN4A*	1762A > G	I588V	0	0	PS3 + PS4(P) + PM2 + PP3 + PP4	LP
*SCN4A*	2015G > A	R672H	0	0	PS3 + PS4 + PM1 + PM2 + PM5(S) + PP1 + PP3 + PP4	P
*SCN4A*	2014C > G	R672G	0	0	PS3 + PS4 + PM1 + PM2 + PM5(S) + PP1 + PP3 + PP4	P
*SCN4A*	2111C > T	T704M	0	0	PS3 + PS4 + PM2 + PP3 + PP4	P
*SCN4A*	2638_2640del	K880del	0.000729	0.001976	PS3 + PS4(P) + PM4 + PP4	LP
*SCN4A*	3404G > A	R1135H	0	0	PS3 + PS4 + PM1 + PM2 + PM5 + PP3 + PP4	P
*SCN4A*	3445G > T	V1149L	0	0	PS4(M) + PM2 + PP3 + PP4	LP
*SCN4A*	4352G > A	R1451H^†^	0	0	PS4(P) + PM1 + PM2 + PM5(S) + PP1 + PP3 + PP4	P
*SCN4A*	4774A > G	M1592V	0	0	PS3 + PS4 + PM2 + PP3 + PP4	P
*SCN4A*	4937C > A	T1646N^†^	0.0001111	0.000504	PS4(P) + PP3 + PP4	VUS
*KCNJ2*	199C > T	R67W	0	0	PS3 + PS4 + PM2 + PM5 + PP3 + PP4	P
*KCNJ2*	334G > T	D112Y^†^	0	0	PS4(P) + PM2 + PP3 + PP4	VUS
*KCNJ2*	637C > T	R213^*^^†^	0	0	PS4(P) + PM2 + PM4 + PP3 + PP4	LP
*KCNJ2*	839A > G	Y280C^†^	0	0	PS4(P) + PM2 + PP3 + PP4	VUS
*KCNJ2*	934C > T	R312C	0	0	PS4 + PM2 + PM5 + PP3 + PP4	P
*KCNJ2*	935G > A	R312H	0	0	PS3 + PS4(M) + PM2 + PM5 + PP3 + PP4	P

† Novel variant;

* Stop codon; PS, strong pathogenic; PM, moderate pathogenic; PP, supporting pathogenic; P, pathogenic; LP, likely pathogenic; VUS, variant with unknown significance.

**Figure 2:**
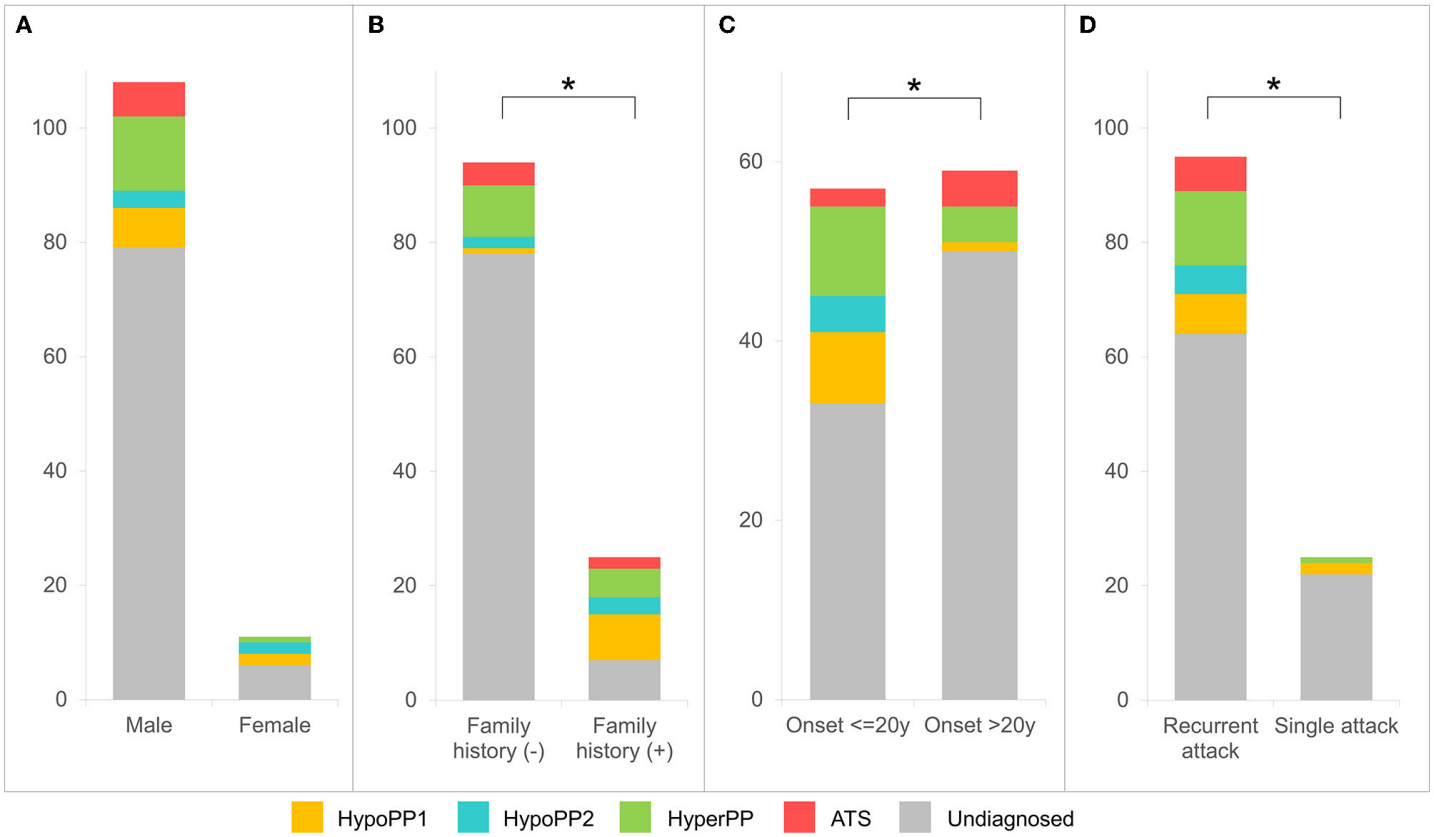
Clinical and molecular diagnostic rate analyses for patients with PP. Molecular diagnostic rates are observed higher in female patients [**(A)** *p* > 0.05], patients with positive family history [**(B)** *p* < 0.0001], with onset age ≤20 years [**(C)** *p* < 0.01], or patients who experienced recurrent paralytic attacks [**(D)** *p* < 0.05].

The molecular diagnostic percentage of patients with hypoPP and hyperPP was 26.2 (17/65) and 32.7% (17/52), respectively (Figure 1C). The percentage between male and female patients was 29/108 and 5/11 (*p* > 0.05) (Figure 2A). Patients with FPP (18/25) were found easier to receive a genetic diagnosis than patients with SPP (16/94) (*p* < 0.0001) (Figure 2B). Pedigree sequencing of other affected/unaffected family members was available from seven pedigrees with FPP, and co-segregation of variants was verified from all but the pedigree carrying p.V1149L variant (LP) in *SCN4A*. Within this pedigree, the same variant was detected from the asymptomatic mother of the proband as well, suggesting a lower penetrance in female (Supplementary Table 2).

A higher diagnostic rate was observed in patients with onset age ≤20 years than that of later onset (>20 years), at 24/57 vs. 9/59 (*p* < 0.01) (Figure 2C). Otherwise, the positive rate of patients with recurrent paralytic attacks (31/94) was detected as higher than those who experienced only a single attack (3/25) (*p* < 0.05) (Figure 2D).

### 3.3. *CACNA1S* variants

**Figure 3:**
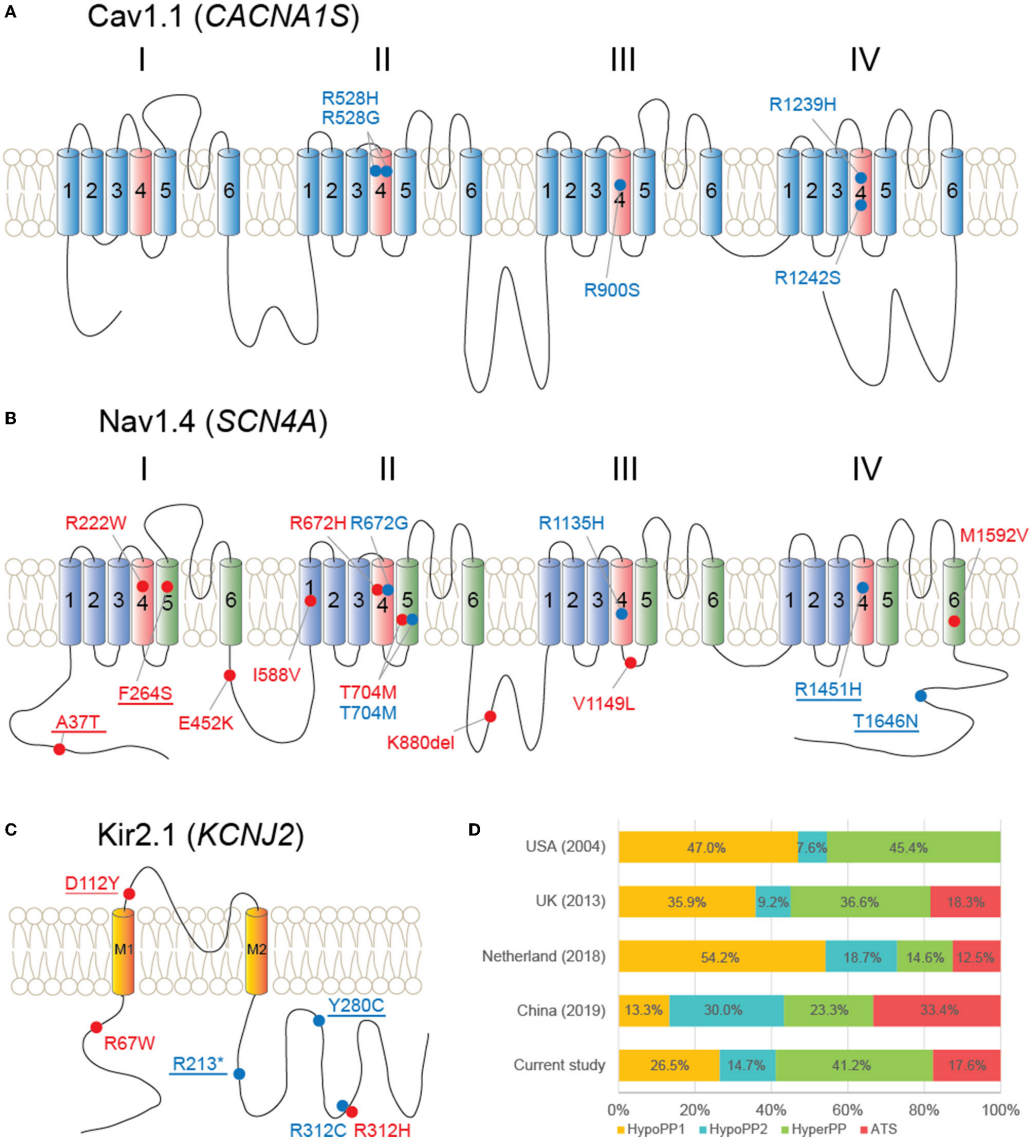
Schematic diagrams of proteins and variants detected in this study and literature review. **(A)** Cav1.1 protein and five variants locate at its voltage–sensor domains (light red color). **(B)** Nav1.4 and 14 variants scatter throughout the protein. **(C)** Kir2.1 and location of six variants. Red dot and label: hyperPP phenotype; blue dot and label: hypoPP phenotype; underline: novel variants. **(D)** Literature review of genetic proportions from large-group studies of multiple countries, covering all *CACNA1S, SCN4A*, and *KCNJ2* genes.

Within nine patients with hypoPP, we found five initially reported variants within the *CACNA1S* gene, comprising p.R528H (five cases; P), p.R528G (one case; P), p.R900S (one case; P), p.R1239H (one case; P), and p.R1242S (one case; LP). All of these variants are located in voltage–sensor domains of the CaV1.1 protein (Figure 3A).

### 3.4. *SCN4A* variants

From five patients with hypoPP, five distinct *SCN4A* variants were detected, including p.R672G (P), p.T704M (P), p.R1135H (P), p.R1451H (novel; P), and p.T1646N (VUS). Therein, p.T1646N could also be discovered from gnomAD_EAS (allele frequency = 0.0001) and jMorp databases (allele frequency = 0.0005; odds ratio = 8.37, *p* = 0.0362).

*SCN4A* variants were found in 14 patients clinically suspected with hyperPP as well, including p.A37T (three cases; novel; VUS), p.R222W (two cases; P), p.F264S (one case; novel; VUS), p.E452K (one case; VUS), p.R672H (one case; P), p.T704M (two cases; P), p.K880del (one case; LP), p.V1149L (one case; LP), and p.M1592V (one case; P) (Figure 3B). Within the four VUS variants, p.A37T and p.K880del were found on the population databases, with frequencies of 0.0003 and 0.0007 on gnomAD_EAS, and 0.0002 (odds ratio = 52.02, *p* < 0.0001) and 0.002 (odds ratio = 2.13, *p* = 0.45) on jMorp, respectively. Therein, a recent functional analysis of p.K880del revealed a weak functional effect on Nav1.4, increasing the excitability of the sarcolemma, which could represent a potential pathogenic factor (17).

### 3.5. *KCNJ2* variants

In total, six different *KCNJ2* variants were found in six patients with either hypoPP (three cases) or hyperPP (three cases) phenotype. The variants were p.R67W (P), p.D112Y (novel; VUS), p.R213^*^ (novel; LP), p.R280C (novel; VUS), p.R312C (P), and p.R312H (P) (Figure 3C). There was no discernible skeletal deformity in any of these patients. In four cases, electrocardiogram (ECG) data were available, and none of them revealed ventricular arrhythmias.

## 4. Discussion

In this study, based on our relaxed enrollment criteria, we collected 119 unrelated index patients with clinically suspected PP, and the genetic diagnosis was only procured from 28.6% of them. However, even using the more stringent criteria involving recurrent paralytic attacks, the diagnostic rate was 33.0% (31/94). Both rates were significantly lower than previous studies conducted in the USA and China (11, 12). In terms of hypoPP, the diagnostic rate was 26.2%, which was also much lower than in several western countries (64.3–89.2%) (12, 18–20), but higher than in a Taiwan study (12.5%) (21) (Table 2). In contrast, 18 out of 25 (72.0%) patients with FPP received a molecular diagnosis, showing a much higher yield than patients with SPP (17.0%; *p* < 0.0001). Otherwise, patients with early onset (≤20 years) were found to be more amenable to molecular diagnosis than that of late-onset cases (24/57 vs. 9/59).

**Table 2:** Literature review of large-group studies for patients with PP.

**Reference**	**HypoPP1**	**HypoPP2**	**HyperPP**	**ATS**	**Total**	**Rate**	**M:F**
Sternberg et al. (20) (France; pedigree)	40/58	5/58	/	/	45/58	77.6%	/
Miller et al. (12) (USA; pedigree)	31/56	5/56	30/47	0	66/103	64.1%	1.6:1
Matthews et al. (19) (USA; case)	65/83	9/83	/	/	74/83	89.2%	/
Sung et al. (21) (Taiwan; pedigree)	3/64	5/64	/	/	3/64	12.5%	63:1
Horga et al. (1) (UK; pedigree)	47	12	48	24	131	/	2.3:1
Stunnenberg et al. (2) (Netherlands; pedigree)	26	9	7	6	48	/	/
Luo et al. (11) (China; pedigree)	4	9	7	10	30/53	56.6%	7.8:1
Sasaki et al. (5) (Japan; pedigree)	16	12	11	/	39	/	/
Brugnoni et al. (18) (Italy etc. case)	38/59	12/59	/	/	50/59	84.8%	2.5:1
Current study	9/65	5/65	14/52	6	34/119	28.6%	9.8:1

ATS, Andersen-Tawil syndrome; M, male; F, female; /, not available.

Taken together, the aforementioned differences between present and previous studies may be contributed by but not limited to a high proportion of late-onset PP (59/116) and SPP (94/119) in our case series. This is comparable to the Taiwan study with by far the lowest diagnostic rate, where 93.8% (60/64) of their cases were SPP (21). Furthermore, we also noted that the diagnostic rates of PP were concurrently lower in Asia than that of studies in Europe or the USA, and thus, a racial difference should be taken into account as well.

When compared to multiple large-group studies that covered all *CACNA1S, SCN4A*, and *KCNJ2* genes, the genetic proportion of our diagnosed patients had the following characteristics: (1) hypoPP1 was more common than hypoPP2 (9:5), which was comparable to a recent Japanese study (4:3) (5), but not as noticeable as the difference observed in western countries; (2) hyperPP (41.2%) was more common than either hypoPP phenotype alone (Figure 3D). Our case series included 108 men and 11 women, for a gender ratio of about 10:1. This gender disparity could be explained by females' lower penetrance, which is consistent with previous findings (18, 22, 23). Though without substantial variation, female patients (45.5%) were more likely than male patients (26.9%) to receive a molecular diagnosis.

All five *CACNA1S* gene variants were found at arginines of S4 voltage sensors in domains II (p.R528H/G), III (p.R900S), and IV (p.R1239H, p.R1242S) of Cav1.1. Functional assessments have been conducted for all these variants except p.R1242S. Reduced amplitude of inward Ca^2+^ currents was observed from all of the four variants, and an abnormal gating pore leak current was detected from p.R528H/G and p.R1239H (24–26). These changes would result in susceptibility to recurrent episodes of depolarization-induced loss of excitability and weakness in HypoPP (27).

Unlike *CACNA1S, SCN4A* variants associated with both hyperPP and hypoPP2 were found throughout the protein Nav1.4. It is of note that multiple *SCN4A* variants, previously reported from patients with hypoPP (p.R222W and p.R672H) or non-dystrophic myotonia (p.E452K) (19, 28, 29), developed hyperPP phenotype in our patients. These findings, together with the p.T704M variant, which is associated with both hyperPP and hypoPP2 phenotypes in the present study, highlight the phenotypic heterogeneity of sodium channelopathies. Mutations associated with hyperPP produce the gain-of-function changes for Nav1.4, commonly exhibiting defects of fast and/or slow inactivation, and occasionally showing an enhancement of activation (27). In contrast, multiple mechanisms have been elucidated from hypoPP2 mutations, consisting of loss-of-function changes of Nav1.4, such as enhanced inactivation and decoupling of voltage-sensor displacement to channel opening (29, 30), as well as the gating pore “leakage” current (31). Reduced Nav1.4 currents may also contribute to the reduced excitability of the muscle membrane, leading to paralysis. Among novel *SCN4A* variants (p.A37T, p.F264S, p.R1451H, and p.T1646N), p.A37T and p.T1646N locate at cytoplasmic N or C terminus of Nav1.4, the domains where multiple variants have been reported, and a p.F1705I variant was found causing fast inactivation defects (32).

Despite PP, ventricular arrhythmias, and dysmorphism being identified as the triad of ATS, patients frequently lack one or more features of the classic triad. As demonstrated in our patients, among all six patients carrying *KCNJ2* variants, none of them showed any noticeable dysmorphic features or electrocardiographic abnormalities. This PP-only phenotype complicates the clinical diagnosis of ATS and emphasizes the importance of genetic screening for the *KCNJ2* gene in patients with isolated PP. Our outcomes also indicate that the frequency of ATS with PP-only phenotype may be underestimated, referring to a previous report in Japan (2/57) (33). Mutations of *KCNJ2* locate throughout the Kir2.1 protein, and cellular analyses revealed the loss-of-function and mostly with a dominant-negative effect on lowering the inward rectifier current, which subsequently depolarizes resting membrane potential and leads to paralysis (10, 34).

On the other hand, SPP is predominant in our case series (94:25), and the etiology requires further research. Recently, within a molecularly undiagnosed Japanese SPP cohort, disease susceptibility was confirmed for nine single-nucleotide variants (SNVs), discovered in genome-wide association studies from SPP and/or thyrotoxic PP in Asian populations (35–40). All of these SNVs are found on chromosome 17 downstream of the *KCNJ2* gene, with strong linkage disequilibrium, implying a genetic basis for the undiagnosed SPP.

In this study, we adopted a relatively broad inclusion criterion and obtained a low diagnostic rate (28.6%) from a case series of patients with PP. As indicated in our subsequent statistical analyses, the diagnostic yield could be improved using a more stringent enrollment criterion, such as positive family history, early-onset, and recurrent paralysis. However, since disease-associated variants were also identified from sporadic, atypical, or first-onset cases, we decided to involve all of them in this study. We could not exclude the possibility that the part of our patients was actually not PP, particularly those patients with hyperPP. Another limitation of this study is that the pathogenicity of the VUS variants has not been functionally verified, whereas the possible existence of benign variants would make the diagnostic rate even lower.

In summary, we evaluate the low molecular diagnostic rate and specific genetic proportion of a large Japanese case series of patients suspected of PP. Our outcomes outline the racial diversity and etiological complexity of patients with PP in Japan. Future research should attempt to explore other possible causes of undiagnosed PP, the pathogenicity of detected variants in known PP disease-causing genes, particularly VUS variants, and the pathogenesis of SPP-associated SNVs.

## Data availability statement

The datasets presented in this article are not readily available because of ethical and privacy restrictions. Requests to access the datasets should be directed to the corresponding author.

## Ethics statement

The studies involving human participants were reviewed and approved by Institutional Review Board of Kagoshima University (Application ID: 490). Written informed consent to participate in this study was provided by the participants' legal guardian/next of kin.

## Author contributions

HT conceptualized the study. J-HY and AY conducted the genetic experiments and analyzed the data. J-HY, YujH, AH, MA, TN, YuH, and YS participated in the clinical data acquisition and analysis. J-HY drafted the original manuscript. All authors revised the manuscript and approved the final version.
