# Supplementary material for: Gene panel analysis of 119 index patients with suspected periodic paralysis in Japan
Source: Front Neurol. 2023 Jan 26;14:1078195. doi: 10.3389/fneur.2023.1078195 (PMC9908745; doi:10.3389/fneur.2023.1078195)
Supplement: Supplementary file 1 [file Data_Sheet_1.PDF]

Supplementary table 1 Modified ACMG/AMP classification

Evidence class	ACMG criteria	Comment	Classification
PVS1	Null variant (nonsense, frameshift, canonical +/− 1 or 2 splice sites, initiation codon, single or multi-exon deletion) in a gene where loss of function (LOF) is a known mechanism of disease	Loss of function (LOF) and/or haploinsufficiency has not been clearly identified as disease mechanisms for *CACNA1S/SCN4A/KCNJ2* heterozygous variants	Not applicable
PS1	Same amino acid change as a previously established pathogenic variant regardless of nucleotide change	Follows primary definition but none of the variants meet this criterion	Not applicable
PS2/PM6	PS2: De novo (both maternity and paternity confirmed) in a patient with the disease and no family history<br>PM6: Assumed de novo, but without confirmation of paternity and maternity	Follows primary definition but none of the variants meet this criterion	Not applicable
PS3	Well-established in vitro or in vivo functional studies strongly supportive of a damaging effect on gene or gene product	Functional effect validated using in vitro electrophysiological studies	PS3
PS4	The prevalence of the variant in affected individuals is significantly increased compared to the prevalence in controls	Refers to "ClinGen's RASopathy Expert Panel consensus methods for variant interpretations"	PS4: ⩾5 independent occurrences<br>PS4(M): 3~4 independent occurrences<br>PS4(P): 1~2 independent occurrences
PM1	Located in a mutational hot spot and/or critical and well-established functional domain (e.g. active site of an enzyme) without benign variation	Variants locate at voltage sensor domain of Cav1.1 and Nav1.4 meet this criterion	PM1
PM2	Absent from controls (or at extremely low frequency if recessive) in Exome Sequencing Project, 1000 Genomes or ExAC	Completely absent in both East Asian population of gnomAD and jMorp databases	PM2
PM3	For recessive disorders, the variant is detected in trans with a pathogenic variant.	Follows primary definition but none of the genes meet this criterion	Not applicable
PM4	Protein length changes as a result of in-frame deletions/insertions in a nonrepeat region or stop-loss variants	Follows primary definition	PM4
PM5	Novel missense change at an amino acid residue where a different missense change determined to be pathogenic has been seen before	Refers to "ClinGen's RASopathy Expert Panel consensus methods for variant interpretations"	PM5(S): ⩾2 different pathogenic missense changes<br>PM5: 1 different pathogenic missense change
PP1	Co-segregation with disease in multiple affected family members in a gene definitively known to cause the disease	Refers to "ClinGen's RASopathy Expert Panel consensus methods for variant interpretations"	PP1(S): ⩾7 meioses<br>PP1(M): 5~6 meioses<br>PP1: 3~4 meioses

PP2	Missense variant in a gene that has a low rate of benign missense variation and in which missense variants are a common mechanism of disease	Follows primary definition but none of these genes meet the criterion	Not applicable
PP3	Multiple lines of computational evidence support a deleterious effect on the gene or gene product (conservation, evolutionary, splicing impact, etc.)	Follows primary definition	PP3
PP4	Patient's phenotype or family history is highly specific for a disease with a single genetic etiology	Periodic paralysis is considered as a specific phenotype for patients with *CACNA1S/SCN4A/KCNJ2* variants	PP4
PP5	Reputable source recently reports variant as pathogenic but the evidence is not available to the laboratory to perform an independent evaluation	Currently, there are no resources that are acceptable for this criterion	Not applicable
