# Supplementary material for: Gene panel analysis of 119 index patients with suspected periodic paralysis in Japan
Source: Front Neurol. 2023 Jan 26;14:1078195. doi: 10.3389/fneur.2023.1078195 (PMC9908745; doi:10.3389/fneur.2023.1078195)
Supplement: Supplementary file 2 [file Data_Sheet_2.PDF]

Supplementary table 2 Clinical features of 119 index patient with suspected periodic paralysis

Patient ID	Clinical diagnosis	P/LP/VUS variant	Subgroup	Gender	Onset age <=20	Recurrent paralytic attack	Positive Family history	Other affected members	Pedigree study
PP001	HyperPP	-	Undiagnosed	M	Y	Y	Y	Father	/
PP002	HypoPP	*CACNA1S* : c.3716G>A, p.R1239H	HypoPP1	M	Y	Y	N	-	/
PP003	HypoPP	-	Undiagnosed	M	Y	Y	Y	Sister	/
PP004	HyperPP	*SCN4A* : c.109G>A, p.A37T	HyperPP	M	N	N	N	-	/
PP005	HyperPP	*SCN4A* : c.109G>A, p.A37T	HyperPP	M	N	Y	N	-	/
PP006	HyperPP	*SCN4A* : c.2111C>T, p.T704M	HyperPP	M	Y	Y	Y	Son	/
PP007	HyperPP	*SCN4A* : c.791T>C, p.F264S	HyperPP	M	Y	Y	N	-	/
PP008	HyperPP	-	Undiagnosed	M	N	Y	N	-	/
PP009	HypoPP	-	Undiagnosed	M	N	Y	N	-	/
PP010	HypoPP	-	Undiagnosed	M	N	N	N	-	/
PP011	HypoPP	-	Undiagnosed	M	Y	Y	N	-	/
PP012	HypoPP	-	Undiagnosed	M	N	Y	N	-	/
PP013	HyperPP	-	Undiagnosed	M	N	Y	N	-	/
PP014	HypoPP	-	Undiagnosed	M	N	N	N	-	/
PP015	HypoPP	-	Undiagnosed	M	N	N	N	-	/
PP016	HypoPP	-	Undiagnosed	M	N	Y	N	-	/
PP017	/	-	Undiagnosed	M	Y	Y	N	-	/
PP018	HypoPP	-	Undiagnosed	M	N	Y	N	-	/
PP019	HypoPP	-	Undiagnosed	M	Y	Y	N	-	/
PP020	HyperPP	*SCN4A* : c.664C>T, p.R222W	HyperPP	M	N	Y	N	-	/
PP021	HyperPP	*SCN4A* : c.664C>T, p.R222W	HyperPP	M	Y	Y	Y	Niece	Niece (+)
PP022	HypoPP	-	Undiagnosed	M	Y	Y	N	-	/
PP023	HypoPP	-	Undiagnosed	M	N	Y	N	-	/
PP024	HypoPP	-	Undiagnosed	M	N	Y	N	-	/
PP025	HyperPP	-	Undiagnosed	M	Y	N	N	-	/
PP026	HypoPP1	*CACNA1S* : c.3726G>T, p.R1242S	HypoPP1	M	Y	N	Y	Father	/
PP027	HyperPP	-	Undiagnosed	M	Y	Y	N	-	/
PP028	HyperPP	-	Undiagnosed	M	Y	Y	N	-	/
PP029	HypoPP	*CACNA1S* : c.1583G>A, p.R528H	HypoPP1	F	N	Y	Y	Father, son	/
PP030	HyperPP	-	Undiagnosed	M	N	Y	N	-	/
PP031	HyperPP	-	Undiagnosed	M	Y	Y	N	-	/
PP032	HyperPP	*KCNJ2* : c.199C>T, p.R67W	ATS	M	N	Y	N	-	/
PP033	HyperPP	*SCN4A* : c.109G>A, p.A37T	HyperPP	M	Y	Y	Y	Father	/
PP034	HypoPP	-	Undiagnosed	F	N	N	N	-	/
PP035	HypoPP	*CACNA1S* : c.2700G>C, p.R900S	HypoPP1	F	Y	Y	Y	Grandmother, father	/
PP036	HypoPP	-	Undiagnosed	M	N	Y	N	-	/
PP037	HypoPP	*SCN4A* : c.4352G>A, p.R1451H	HypoPP2	M	Y	Y	Y	Father, uncle	Father (+), uncle (+)
PP038	HypoPP	-	Undiagnosed	M	N	Y	N	-	/
PP039	HyperPP	-	Undiagnosed	M	N	Y	N	-	/
PP040	HypoPP	-	Undiagnosed	M	N	Y	N	-	/
PP041	HypoPP	-	Undiagnosed	M	N	Y	N	-	/
PP042	HypoPP	-	Undiagnosed	M	N	N	N	-	/
PP043	HypoPP	*CACNA1S* : c.1583G>A, p.R528H	HypoPP1	M	Y	Y	Y	Mother, brother	/
PP044	HyperPP	*SCN4A* : c.1354G>A, p.E452K	HyperPP	M	N	Y	N	-	/
PP045	HypoPP	-	Undiagnosed	M	N	Y	N	-	/
PP046	HyperPP	-	Undiagnosed	M	/	N	N	-	/
PP047	HypoPP	*CACNA1S* : c.1583G>A, p.R528H	HypoPP1	M	Y	Y	Y	Father	/
PP048	HyperPP	-	Undiagnosed	M	Y	Y	N	-	/
PP049	HypoPP	-	Undiagnosed	M	N	Y	N	-	/
PP050	HyperPP	-	Undiagnosed	M	Y	Y	N	-	/
PP051	HyperPP	-	Undiagnosed	M	N	Y	N	-	/
PP052	HypoPP	*CACNA1S* : c.1583G>A, p.R528H	HypoPP1	M	Y	Y	Y	Grandmother, mother	/
PP053	HypoPP	-	Undiagnosed	M	N	Y	N	-	/
PP054	HypoPP	*KCNJ2* : c.934C>T, p.R312C	ATS	M	N	Y	N	-	/
PP055	HypoPP	-	Undiagnosed	M	N	Y	N	-	/
PP056	HyperPP	-	Undiagnosed	M	Y	N	N	-	/
PP057	HypoPP	-	Undiagnosed	M	N	N	N	-	/
PP058	HypoPP	-	Undiagnosed	M	N	N	N	-	/
PP059	HypoPP	-	Undiagnosed	M	N	Y	N	-	/
PP060	HypoPP	-	Undiagnosed	M	N	N	N	-	/
PP061	HypoPP	-	Undiagnosed	M	N	N	N	-	/
PP062	HyperPP	*SCN4A* : c.2015G>A, p.R672H	HyperPP	F	Y	Y	Y	Father, brother, 2 sons	Sons (+)
PP063	HypoPP	-	Undiagnosed	M	Y	Y	N	-	/
PP064	HypoPP	-	Undiagnosed	M	N	Y	N	-	/
PP065	HypoPP	*SCN4A* : c.3404G>A, p.R1135H	HypoPP2	M	Y	Y	N	-	/
PP066	HyperPP	-	Undiagnosed	M	/	N	N	-	/
PP067	HyperPP	*SCN4A* : c.3445G>T, p.V1149L	HyperPP	M	Y	Y	N	-	Father (-), mother (+)
PP068	HyperPP	*SCN4A* : c.2638_2640del, p.K880del	HyperPP	M	Y	Y	N	-	/
PP069	HypoPP	*CACNA1S* : c.1583G>A, p.R528H	HypoPP1	M	Y	N	Y	Grandmother, mother	/
PP070	HypoPP	-	Undiagnosed	M	N	Y	N	-	/
PP071	HyperPP	-	Undiagnosed	M	N	Y	N	-	/
PP072	HyperPP	-	Undiagnosed	M	N	Y	N	-	/
PP073	HyperPP	-	Undiagnosed	M	N	Y	N	-	/
PP074	HypoPP	-	Undiagnosed	M	N	N	N	-	/
PP075	HyperPP	-	Undiagnosed	M	N	Y	N	-	/
PP076	HypoPP	*SCN4A* : c.4937C>A, p.T1646N	HypoPP2	F	/	Y	N	-	/
PP077	HypoPP	-	Undiagnosed	M	Y	Y	N	-	/
PP078	HyperPP	-	Undiagnosed	M	N	Y	N	-	/
PP079	HyperPP	-	Undiagnosed	M	N	Y	N	-	/
PP080	HypoPP	-	Undiagnosed	F	N	Y	N	-	/
PP081	HypoPP	-	Undiagnosed	M	N	N	N	-	/
PP082	HypoPP	*SCN4A* : c.2014C>G, p.R672G	HypoPP2	M	Y	Y	Y	Father, sister	Father (+), sister (+)
PP083	HyperPP	-	Undiagnosed	M	N	Y	N	-	/
PP084	HyperPP	*SCN4A* : c.4774A>G, p.M1592V	HyperPP	M	Y	Y	N	-	/
PP085	HypoPP	-	Undiagnosed	M	Y	Y	N	-	/
PP086	HyperPP	-	Undiagnosed	M	N	Y	N	-	/
PP087	HypoPP	-	Undiagnosed	M	Y	Y	N	-	/
PP088	HypoPP	-	Undiagnosed	F	N	Y	Y	Father	/
PP089	HypoPP	-	Undiagnosed	M	N	N	Y	Father	/
PP090	HyperPP	-	Undiagnosed	M	N	Y	N	-	/
PP091	HypoPP	*KCNJ2* : c.637C>T, p.R213*	ATS	M	N	Y	N	-	/
PP092	HyperPP	-	Undiagnosed	M	Y	Y	N	-	/
PP093	HyperPP	*SCN4A* : c.1762A>G, p.I588V	HyperPP	M	Y	Y	N	-	Brother (-)

PP094	HypoPP	-	Undiagnosed	M	N	Y	Y	Brother	/
PP095	HyperPP	*KCNJ2* : c.935G>A, p.R312H	ATS	M	Y	Y	Y	Father	/
PP096	HypoPP	-	Undiagnosed	M	Y	Y	N	-	/
PP097	HypoPP	*SCN4A* : c.2111C>T, p.T704M	HypoPP2	F	Y	Y	Y	Grandmother, father	/
PP098	/	-	Undiagnosed	F	Y	N	N	-	/
PP099	HypoPP	-	Undiagnosed	M	Y	Y	N	-	/
PP100	HyperPP	*SCN4A* : c.2111C>T, p.T704M	HyperPP	M	Y	Y	Y	Mother, siblings	/
PP101	HyperPP	-	Undiagnosed	M	N	Y	N	-	/
PP102	HypoPP	-	Undiagnosed	M	Y	N	N	-	/
PP103	HyperPP	-	Undiagnosed	M	Y	Y	N	-	/
PP104	HypoPP	-	Undiagnosed	M	N	Y	Y	Father	/
PP105	HypoPP	*KCNJ2* : c.839A>G, p.Y280C	ATS	M	N	Y	N	-	/
PP106	HypoPP	-	Undiagnosed	M	N	Y	N	-	/
PP107	HypoPP	*CACNA1S* : c.1582C>G, p.R528G	HypoPP1	M	Y	Y	Y	Father	Father (+)
PP108	HyperPP	-	Undiagnosed	F	Y	Y	N	-	/
PP109	HyperPP	-	Undiagnosed	M	Y	Y	N	-	/
PP110	HyperPP	*KCNJ2* : c.334G>T, p.D112Y	ATS	M	Y	Y	Y	Father	/
PP111	HypoPP	-	Undiagnosed	M	N	Y	N	-	/
PP112	HyperPP	-	Undiagnosed	F	Y	Y	N	-	/
PP113	HyperPP	-	Undiagnosed	M	Y	Y	N	-	/
PP114	HyperPP	-	Undiagnosed	M	Y	Y	N	-	/
PP115	HypoPP	-	Undiagnosed	M	Y	N	N	-	/
PP116	HypoPP	-	Undiagnosed	M	Y	N	N	-	/
PP117	HyperPP	-	Undiagnosed	M	Y	N	N	-	/
PP118	HyperPP	-	Undiagnosed	M	Y	N	Y	Brother	/
PP119	HypoPP	-	Undiagnosed	M	Y	Y	N	-	/

M: male; F: female; Y: yes; N: no; +: positive; -: negative; /: not available or no data.
